# Identification and Characterization of a Novel Recurrent *ERCC6* Variant in Patients with a Severe Form of Cockayne Syndrome B

**DOI:** 10.3390/genes12121922

**Published:** 2021-11-29

**Authors:** Khouloud Zayoud, Ichraf Kraoua, Asma Chikhaoui, Nadège Calmels, Sami Bouchoucha, Cathy Obringer, Clément Crochemore, Dorra Najjar, Sinda Zarrouk, Najoua Miladi, Vincent Laugel, Miria Ricchetti, Ilhem Turki, Houda Yacoub-Youssef

**Affiliations:** 1 Laboratory of Biomedical Genomics and Oncogenetics (LR16IPT05), Institut Pasteur de Tunis, Université Tunis El Manar, El Manar I, Tunis 1002, Tunisia; zayoudkhouloud@gmail.com (K.Z.); asma.lfsv@gmail.com (A.C.); dorra.najjar2@gmail.com (D.N.); 2 Faculté des Sciences de Bizerte, Bizerte 7000, Tunisia; 3 LR18SP04 and Department of Child and Adolescent Neurology, National Institute Mongi Ben Hmida of Neurology, Tunis 1007, Tunisia; kraoua_ichraf@yahoo.fr (I.K.); ilhem.benyoussef58@gmail.com (I.T.); 4 Laboratoires de Diagnostic Génétique, Institut de Génétique Médicale d’Alsace, Nouvel Hôpital Civil, Hôpitaux Universitaires de Strasbourg, 67000 Strasbourg, France; nadege.calmels@chrustrasbourg.fr; 5 Laboratoire de Génétique Médicale, INSERM U1112, Institut de génétique médicale d’Alsace, CRBS, 67000 Strasbourg, France; obringercathy@gmail.com (C.O.); Vincent.LAUGEL@chru-strasbourg.fr (V.L.); 6 Service Orthopédie, Hôpital d’enfant Béchir Hamza, Tunis 1000, Tunisia; sami.bouchoucha@yahoo.com; 7 Institut Pasteur, Team Stability of Nuclear and Mitochondrial DNA, Stem Cells and Development, UMR 3738 CNRS, 25-28 rue du Dr. Roux, 75015 Paris, France; clement.crochemore@pasteur.fr (C.C.); miria.ricchetti@pasteur.fr (M.R.); 8 Genomics Platform, Institut Pasteur de Tunis (IPT), Tunis-Belvédère, Tunis 1002, Tunisia; sinda.zarrouk@pasteur.tn; 9 Maghreb Medical Center, El Manar III, Tunis 9000, Tunisia; Najoua.miladi@hotmail.fr

**Keywords:** Cockayne syndrome, *ERCC6*, accelerated aging, neurodegeneration, DNA repair disorder

## Abstract

Cockayne syndrome (CS) is a rare disease caused by mutations in *ERCC6*/*CSB* or *ERCC8*/*CSA*. We report here the clinical, genetic, and functional analyses of three unrelated patients mutated in *ERCC6*/*CSB* with a severe phenotype. After clinical examination, two patients were investigated via next generation sequencing, targeting seventeen Nucleotide Excision Repair (NER) genes. All three patients harbored a novel, c.3156dup, homozygous mutation located in exon 18 of *ERCC6*/*CSB* that affects the C-terminal region of the protein. Sanger sequencing confirmed the mutation and the parental segregation in the three families, and Western blots showed a lack of the full-length protein. NER functional impairment was shown by reduced recovery of RNA synthesis with proficient unscheduled DNA synthesis after UV-C radiations in patient-derived fibroblasts. Despite sharing the same mutation, the clinical spectrum was heterogeneous among the three patients, and only two patients displayed clinical photosensitivity. This novel *ERCC6* variant in Tunisian patients suggests a founder effect and has implications for setting-up prenatal diagnosis/genetic counselling in North Africa, where this disease is largely undiagnosed. This study reveals one of the rare cases of CS clinical heterogeneity despite the same mutation. Moreover, the occurrence of an identical homozygous mutation, which either results in clinical photosensitivity or does not, strongly suggests that this classic CS symptom relies on multiple factors.

## 1. Introduction

Cockayne syndrome (CS) is a rare autosomal recessive disorder. It is a progressively devastating disease, which displays multiorgan dysfunction. CS is mainly characterized by psychomotor retardation, cerebral atrophy, microcephaly, mental retardation, sensorineural hearing loss, and premature aging, along with other defects such as cutaneous photosensitivity, kyphosis, ankylosis, and optic atrophy. In 2010, a study highlighted a broad clinical spectrum of 84 cases of CS patients and defined major diagnostic criteria such as microcephaly, as well as minor diagnostic criteria such as photosensitivity [1]. However, these criteria are neither precocious nor specific to CS [2,3]. In fact, most signs and symptoms in CS patients, such as cerebral atrophy, are also found in mitochondrial diseases [4,5]. To complicate the situation, the reported clinical and mutation spectrum of CS keeps expanding [2,6,7].

CS belongs to the family of pathologies related to the NER pathway. NER removes UVs and chemically induced DNA lesions. It includes two distinct sub-pathways: the global genome NER (GG-NER) sub-pathway, a system which prevents mutagenesis by probing the whole genome, and the transcription-coupled NER (TC-NER) which removes transcription-blocking lesions to permit gene expression [8]. Deficiencies in NER proteins can cause a variety of diseases, ranging from ultraviolet radiation-sensitive syndrome (UVSS), to cancer predisposing Xeroderma pigmentosum (XP), to Trichothiodystrophy (TTD), to a severe premature aging disease such as CS.

In Europe, the incidence of CS is estimated to be 1 case per 200,000 births [9]. Although the incidence rates of other DNA repair disorders, such as XP have been reported in Tunisia [10], no epidemiological data are available for CS.

To date, genetic investigations have shown that CS is essentially assigned to mutations in two genes of the TC-NER pathway: *ERCC6* (*CSB*, OMIM 609413) (NM_000124) and *ERCC8* (*CSA*, OMIM 609412) (NM_000082.3). Deep clinical and molecular analyses in a large cohort of patients revealed that phenotype/genotype correlation is difficult to establish, even with a huge heterogeneity among CS patients (including siblings) [1,11,12,13,14]. The most severe cases, CS type II, most often occur due to *CSB* mutations [15], whereas *CSA* mutations are generally associated with the milder form, CS type I [6]. However, this association is not always straightforward, for instance the mild form can also be due to *CSB* mutations. Of note, clinical photosensitivity is detected in only a fraction of patients, displaying a variety of mutations, and is essentially uncoupled from the severity of the disease [2,16]. 

The phenotypic variability represents, therefore, a challenge for establishing a diagnosis and supportive treatment, plus patients exhibit a short life span (generally less than 5 years for the severe forms, and 16 years for the moderate phenotypes) [17]. Hence, next generation sequencing technologies are of particular interest for molecular diagnosis of such cases [18]. 

To date, approximately 65% of CS patients have been linked to mutations in *ERCC6* and 35% to mutation in *ERCC8* [15]. Interestingly, CS patients in Tunisia and other Arab countries carry mutations predominantly in *ERCC8*. Several studies have suggested that the relatively larger proportion of *ERCC8* defects in these areas is attributed to founder effects amplified by high frequencies of consanguineous unions [1,2]. For instance, a high carrier frequency of the p.Tyr322* CS-disease-causing variation in *ERCC8* was reported among Christian Arabs in northern Israel, which is considered as an ancient founder mutation that may have originated in the Christian Lebanese community [19].

To date, seventeen CS patients have been reported in the Tunisian population: two siblings with an independent mutation (c.400-2A>G) in *ERCC8/CSA* [20] and two other siblings with the recurrent mutation (c.598_600delins) *ERCC8/CSA* [21]. Five more CS patients have been clinically and biochemically characterized, but the mutation has not been identified [16,22]. Recently, we reported a detailed clinical characterization of eight CS-A patients. In that cohort, we showed that siblings carrying either the c.843+1G>C or the c. 598_600delinsAA mutations display remarkable phenotypic differences [23].

In the present study, we describe a novel *ERCC6* mutation in three CS patients belonging to independent families that originate from northwestern Tunisia. The targeted gene sequencing showed a homozygous variation in all cases. We showed its deleterious effect on the CSB protein by Western blots and defective repair of UV-induced DNA damage in CS patient cells. 

## 2. Materials and Methods

### 2.1. Patients 

Three patients were recruited from the Department of Child Neurology (National Institute Mongi Ben Hmida of Tunis) in 2018–2019, where they had undergone neurological and general examination routine since 2017. Blood tests, metabolic tests, and CT scans and/or brain MRIs and electrophysiological studies, as well as genetic tests, have been completed for these patients. After obtaining written informed consent from the families of the patients (as these CS patients were minors), blood, skin biopsies, and genealogical data were collected. The present study was conducted in accordance with Helsinki principles and approved by Institute Pasteur Ethics Committee in Tunisia under the ethical accord number (reference 2017/31/I/LR16IPT05/V2).

### 2.2. DNA Extraction, Quantification, and Quality Control 

Genomic DNAs (gDNA) was isolated from whole blood samples of patients and their parents, using a FlexiGene kit (Qiagen) in accordance with the instructions of the manufacturer. DNA concentration and quality were assessed using a Nanodrop Spectrophotometer (Thermo Scientific, Wilmington, NC, USA). The required DNA concentration for NGS analysis was adjusted to 5 µg.

### 2.3. gDNA Sequencing 

Genetic studies were started by screening for the recurrent *ERCC8* pathogenic variation, already described in North African CS patients (NM_000082.3 c. 598_600delinsAA p.Tyr200Lysfs*12) using Sanger sequencing. *ERCC8* exon 7 was amplified with the following primers set (F: 5′ CCCTTTGAACTTATCACCTG 3′ R: 5′ CCTCTGTGTCCCTAGCACAAT 3′) and sequenced using the ABI 3130 Genetic Analyzer (Applied Biosystems).

#### 2.3.1. Targeted Next Generation Sequencing 

In absence of the recurrent variation, molecular screening of two patients was continued by a next generation sequencing (NGS) assay, targeting 17 genes involved in the NER pathway (*DDB2*, *ERCC1*, *ERCC2*, *ERCC3*, *ERCC4*, *ERCC5*, *ERCC6*, *ERCC8*, *GTF2H5*, *MPLKIP*, *PCNA*, *POLH*, *RNF113A*, *SMARCAL1*, *UVSSA*, *XPA*, *XPC*) adapted from [18]. Regions of interest (exons and flanking intronic sequences) were captured using SureSelect QXT Agilent probes, and libraries were sequenced on a NextSeq550 Illumina platform. Single nucleotide variants (SNVs) and indels calling were performed with the Genome Analysis Toolkit v.3.4.46 with the help of an in-house pipeline (STARK, https://github.com/bioinfo-chru-strasbourg/STARK, accessed on 19 May 2019) and following the GATK best practice. Annotation and ranking of SNVs and indels were performed by VaRank [24]. Copy number variants (CNVs) were called using the CANOES program [25] and annotated with AnnotSV [26]. 

#### 2.3.2. Targeted Sanger Sequencing

The polymerase chain reaction (PCR) was performed using primer pair covering exon 18 of *ERCC6* (F: 5’ CTGCAAGACCTGGGAGATT 3’ R: 5’ CAGGGTCTCTTTCAAAGGA 3’). PCR products were sequenced on automated ABI3130 (Applied Biosystems, Waltham, MA, USA) using the ABI Prism BigDye Terminator V3.1 Cycle Sequencing Kit.

### 2.4. DNA Repair Assay in Primary Dermal Fibroblasts

Dermal fibroblasts were obtained from skin biopsies of two patients (CS10 and CS14). Cells were grown at 37 °C in 5% CO_2_ humidified atmosphere in Dulbecco’s Modified Eagle Medium (DMEM) (1 g/L glucose) with GLUTAMAX (Life Technologies (Gibco) supplemented with 10% of fetal calf serum (Gibco) and 1% penicillin/streptomycin (Gibco). All primary fibroblast cultures were assessed at the same passage number, PN (PN 3 to 4). The activity of the NER system following UV irradiations was measured via unscheduled DNA synthesis (UDS) and recovery of RNA synthesis (RRS) after DNA damaging. These two assays are standard tests for the clinical diagnosis of DNA repair-related diseases [27,28,29]. 

Briefly, fibroblasts were plated on coverslips in 6-well plates and exposed to UV-C doses at 0, 5, 10, and 15 J/m^2^. DNA de novo synthesis was evaluated via the incorporation of 5-ethynyl-2′-deoxyuridine (5-EdU) in CS10 and CS14 patient cells, in a healthy donor (negative control), and in an XPF and a CS donor (positive controls). Similarly, and, after 24 h, primary fibroblasts irradiated with UV-C doses (0, 6, 12, and 20 J/m^2^), underwent the RRS assay. The RNA synthesis was performed by evaluating the incorporation of 5-ethynyl uridine (5-EU). The images were analyzed with Image J for 50 randomly selected cells, originating from three independent experiments, and the average nuclear fluorescence intensity was calculated. 

### 2.5. Western Blotting

Cells in culture were pelleted and lysed with lysis buffer (50 mM Tris-HCl pH 7.5, 150 mM NaCl, 1% Triton X-100, 0.1% SDS, 1 mM EDTA), and protease/phosphatase inhibitor mixture (Roche, Basel, Switzerland)). Lysed cells were not centrifuged, and the whole extract was subjected to sonication (Bioruptor, Diagenode, Liège, Belgium). The protein content was determined with the Bradford assay, and 15 μg of protein were boiled in the presence of Laemmli Sample Buffer (#161-0737, Bio-Rad, Hercules, CA, USA) containing 2.5% of 2-mercaptoethanol (#M3148, Sigma-Aldrich, St. Louis, MO, USA) at 95 °C for 5 min, run for SDS-PAGE (4–15% Mini-PROTEAN TGX gel, #4568083, Bio-Rad, Hercules, CA, USA), and transferred onto a nitrocellulose transfer membrane (Trans-Blot Turbo, #1704158, Bio-Rad, Hercules, CA, USA). 

The membrane was then blocked with 5% milk in phosphate buffer saline (PBS)-0.1% Tween20 (#P1379, Sigma-Aldrich, St. Louis, MO, USA) for 1 h at room temperature (RT) and probed with a specific primary antibody, either rabbit polyclonal α-CSB (1:1000, #ab96089, Abcam, Cambridge, UK) that recognizes aa 300–750 or rabbit polyclonal α-CSB (1:2000, #A301-345A, Bethyl, Montgomery, TX, USA) that recognizes aa 1–50, overnight at 4 °C. After five washes in PBS containing 0.1% Tween20, the membrane was incubated with HRP-conjugated secondary antibody (1:10,000, Thermo Fisher Scientific, Waltham, MA, USA), as well as α-GAPDH (1:5000, #12004168, Bio-Rad, Hercules, CA, USA) human Fab fragments for 1h at RT, revealed by chemiluminescence and fluorescence, respectively. Detection was performed using ChemiDoc MP Imaging System (Bio-Rad, Hercules, CA, USA). Staining with ATX Ponceau S Red (#09189, Sigma-Aldrich, St. Louis, MO, USA) was used as a further marker of protein content. Uncropped and unprocessed scans of all the blots can be provided upon request.

## 3. Results

### 3.1. Clinical Features of CS-B Patients 

Clinical, imaging, and genetic characterization of the three patients are summarized in Table 1. 

#### 3.1.1. General Presentation of the Patients

**Figure 1:**
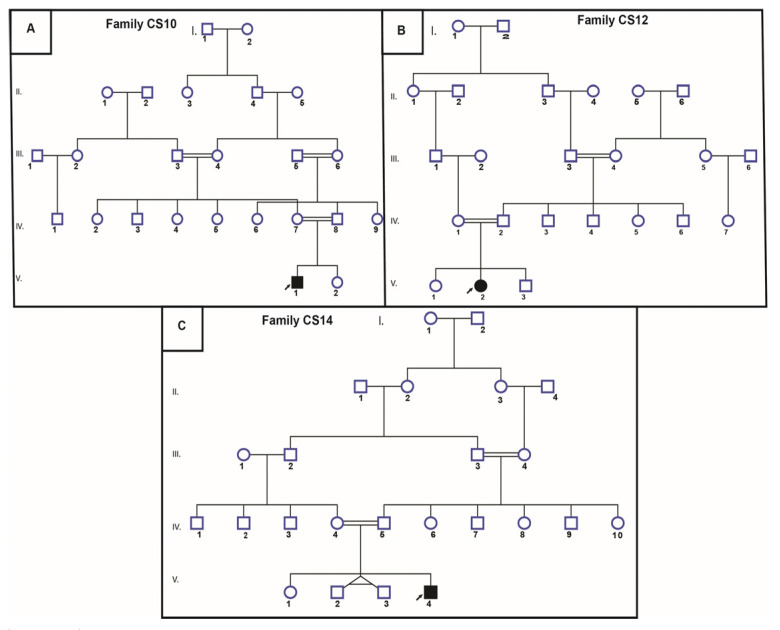
Pedigree of three unrelated Tunisian families. (**A**) pedigree of the CS10 family (**B**) pedigree of the CS12 family (**C**) pedigree of the CS14 family. The studied proband is indicated with an arrow.

This cohort includes two males (CS10 and CS14, aged 2 and 6 years, respectively) and one female (CS12, aged 7 years) from three unrelated Tunisian families. All patients were born from first-degree consanguineous parents, who originated from northwestern Tunisia (Figure 1). 

#### 3.1.2. Pre- and Post-Natal Abnormalities

The reported patients were born at term. Prenatal microcephaly was detected in all three patients through ultrasound screening (Table 1). Postnatally, birth weight was within the normal low range for all patients (mean birth weight of CS patients was 2600 g, ranging from 2300 g to 2950 g); birth height was also normal, whereas the head circumference values were under the normal range (mean head circumference of CS patients was 32.3 cm, with the normal average of the head circumference of a newborn estimated as 35 cm). All three patients developed severe growth failure and microcephaly (mean weight −7 SD, mean head circumference −7.3 SD) (Table 1, and Supplementary Figure S1). 

#### 3.1.3. Behavioral Abnormalities, and Muscular, Neurological, and Neurosensory Problems

All patients arrived in our department for examination displaying a psychomotor delay. Two patients (CS10 and CS12) were able to sit independently at a mean age of 6.5 months, whereas CS14 was unable to do so. Independent walking was acquired late for two patients (CS10 and CS12, at the age of 1.5 and 4 years, respectively) whereas patient CS14 could not walk independently. 

As a general observation, the three patients had no language skills. However, they were interactive. Behavioral disturbances due to irritability and sleep disorders were not reported. Neurological examination showed a spasticity of limbs with predominance in lower limbs in all patients, leading to a progressive flexion retraction and neurological abnormalities in two patients (CS12 and CS14). Ataxia was observed in two cases (CS10 and CS12) and a kyphosis in patient CS14. No patient developed extrapyramidal symptoms. 

Sensorineural deafness was detected in all cases. Ophthalmological examination revealed bilateral cataract for CS10, who underwent eye surgery at the age of 8 months. Pigmentary retinopathy was observed in the CS12 patient and optic atrophy in the CS14 patient (the most severe case). We also reported cryptorchidism in CS10 and CS14 (Table 1).

#### 3.1.4. Facial, Dental, and Skin Anomalies

All patients displayed the typical CS appearance, with enophtalmia, large ears, thin skin, and a bird-like nose. In addition, teeth caries were reported in all patients, and anomalies in tooth shape, size, and number were reported in CS12 and CS14 (CS10 was too young for assessing these parameters).

Clinical photosensitivity was observed in CS10 and CS12, whereas pigmentation abnormalities were observed in patient CS14 (Table 1).

#### 3.1.5. Laboratory Investigations 

The biochemical analysis of AST/ALT showed hepatic cytolysis for all three patients, with the corresponding values of 65/77 for patient CS10 at the age of 2 years, 108/180 for patient CS12 at the age of 7 years, and 45/80 for patient CS14 at the age of 8 years (Table 1). A mild serum creatinine decrease was also noted in all cases (CS10: 22 µmol/L; CS12: 20 µmol/L; CS14: 31 µmol/L), compared to healthy controls (mean secretion level: 80 µmol/L ranging from 50 µmol/L to 110 µmol/L).

#### 3.1.6. Neuroimaging Analysis

**Figure 2:**
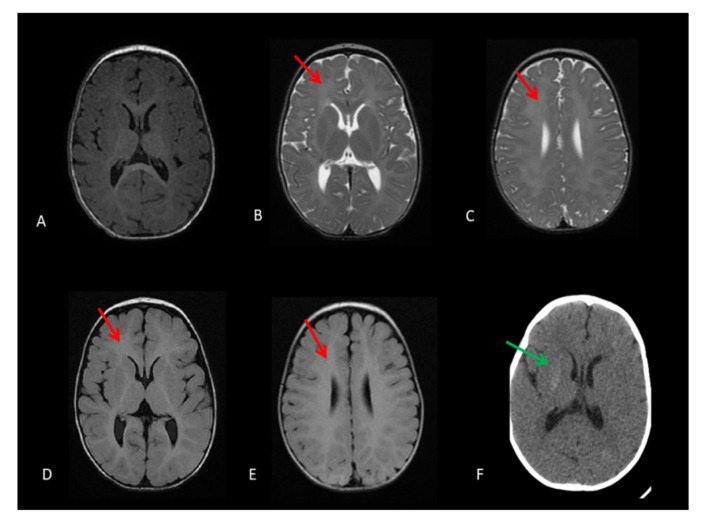
MRI images of patient CS10. (**A**) Axial T1-weighted image, (**B**,**C**) axial T2-weighted images, and (**D**,**E**) axial FLAIR-weighted images, showing isointensity of periventricular white matter on T1 and hyperintensity on T2. FLAIR suggestive of hypomyelinating leukodystrophy (red arrows). (**F**) CT scan shows lenticular calcifications (green arrow).

Magnetic resonance imaging (MRI) showed hypomyelination in two patients (CS10, CS14) but not in CS12 (Figure 2). 

Cerebellar atrophy and brainstem atrophy were observed in all cases. Two out of three patients (CS10, CS14) showed the full table of the features of neurological impairment, typical of CS (Table 1). 

#### 3.1.7. Neurophysiological Studies

Electroneuromyography (ENMG) was performed in two patients (CS12 and CS14). Results were compatible with a sensory-motor polyneuropathy of a demyelinating mechanism [30]. Nerve conduction velocities were studied in two patients (CS12 and CS14), resulting in slow values in both cases (23.5 m/s and 16 m/s, respectively) (Table 1). 

### 3.2. Genetic and Biochemical Analyses

#### 3.2.1. Sanger Sequencing for Recurrent Mutations 

Clinical presentation of the three patients (CS10, CS12 and CS14) suggested Cockayne syndrome diagnosis. Molecular investigations have been started by screening the recurrent *ERCC8* mutation c.598_600delinsAA using targeted Sanger sequencing. Indeed, this frameshift variant, introducing a premature stop codon resulting in a truncated protein, has been previously described in seven Tunisian families [21]. This variant was not found in the patients, and, therefore, samples from two patients underwent targeted gene sequencing covering exons of the 17 genes involved in the NER pathway. 

#### 3.2.2. Targeted Gene Sequencing Finding and Sanger Sequencing Validation for the Novel Variant 

**Figure 3:**
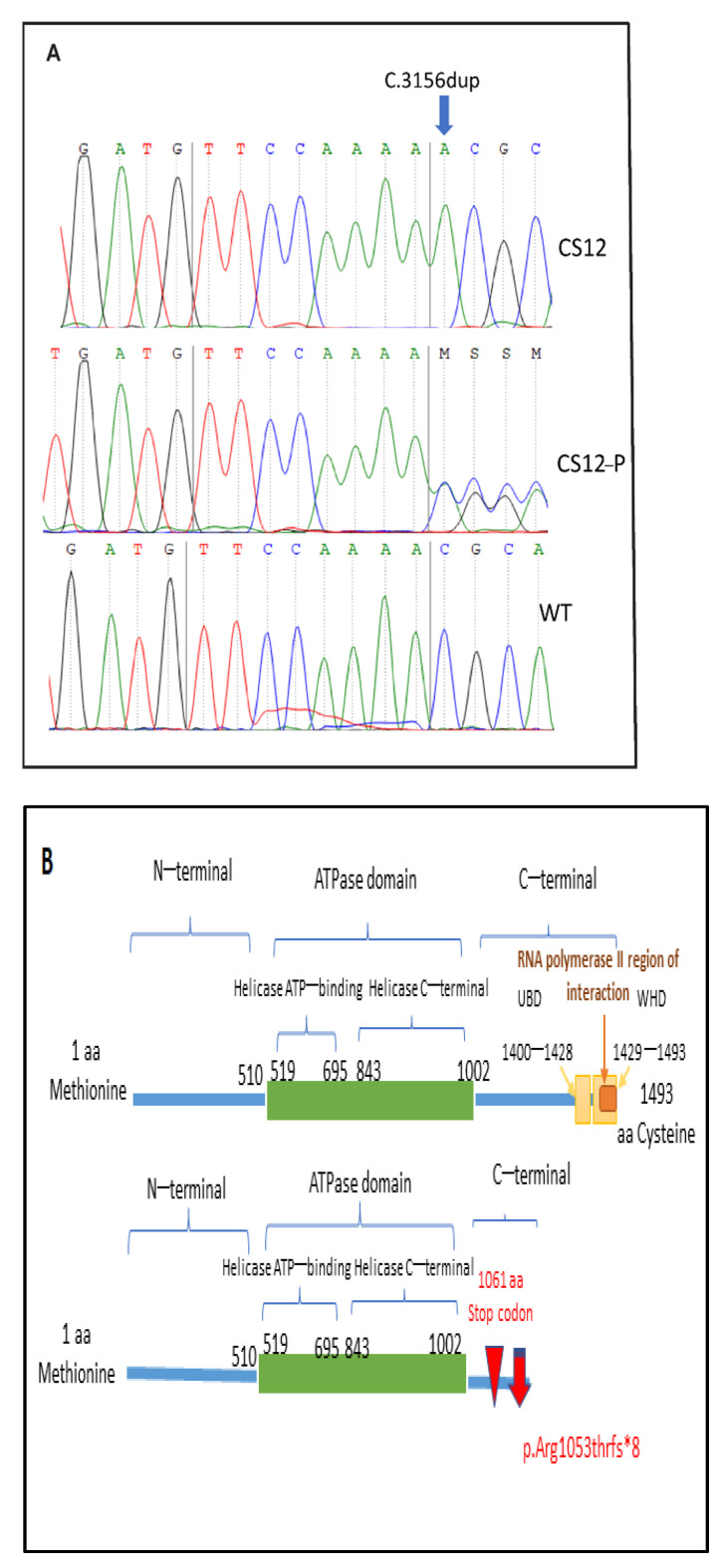
Genetic analysis. (**A**) Electropherogram showing the c.3156dup mutation at homozygous state in CS12, at a heterozygous state in the mother CS12-P, compared to a wild-type sample. (**B**) Schematic representation of the domains in the CSB protein (upper panel) and the novel variant (lower panel). The inverted red triangle represents the frameshift mutation and the red arrow the stop codon 8 aa downstream.

Targeted gene sequencing in two patients (CS10, CS14) identified a novel homozygous variation in exon 18 of the *ERCC6* gene (NM_000124.3). The identified frameshift variation c.3156dup p. (Arg1053Thrfs*8) was then confirmed by Sanger sequencing in the three CS patients (Figure 3A, only patient CS12 is shown). The Sanger sequencing also confirmed the heterozygous carrier status of their respective parents (parents of CS10 and CS14, mother of CS12). This variation has been described only once at the heterozygous state in the gnomAD control database (allele frequency: 0.000003981). This variation causes a reading frameshift and a premature stop codon after eight codons. At the protein level, it could modify the protein sequence at p. (Arg1053Thrfs*8), resulting in a truncated protein (Figure 3B). 

### 3.3. Western Blot Validation of Protein Alteration

**Figure 4:**
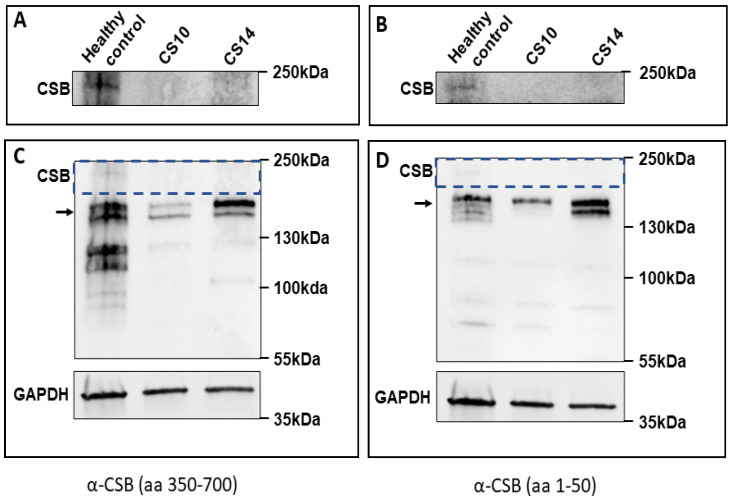
Western blot of CSB. (**A**,**C**) α-CSB rabbit polyclonal Abcam. (**B**,**D**) α-CSB rabbit polyclonal Bethyl. (**A**,**B**): the full-length form of the CSB protein is absent from CS10 and CS14 fibroblasts whereas it is present in a healthy control. (**C**,**D**): larger-sized Western blots at a lower exposure than in (**A**,**B**); arrows indicate the expected position of the CSB-piggyBac fusion protein (which is more abundant than the full-length CSB); hatched rectangles correspond to the part of the blot shown in (**A**,**B**). Lower panels: immunoblot of the loading control GAPDH.

Because of the insertion of a PGBD3 piggyBac transposon into *ERCC6* intron 5, alternative splicing of the human *ERCC6* gene results in the CSB full-length protein (1493 aa) as well as a CSB-PGBD3 fusion protein that joins the N-terminal CSB domain to the C-terminal PGBD3 transposase domain (1061 aa) [31]. Western blot analysis using a CSB antibody that targets aa 300–750, as well as an antibody that targets the N-terminal portion of the protein (aa 1–50), did not show the full-length protein in CS10 and CS14, whereas it was present in control fibroblasts from a healthy donor (Figure 4A,B). The truncated protein resulting from the mutation has an expected length of 1061 aa, and is, therefore, indistinguishable from the CSB PGBD3 fusion protein, also of 1061 aa in length (Figure 4C,D)

### 3.4. Cellular Response to UV in CS Patients

**Figure 5:**
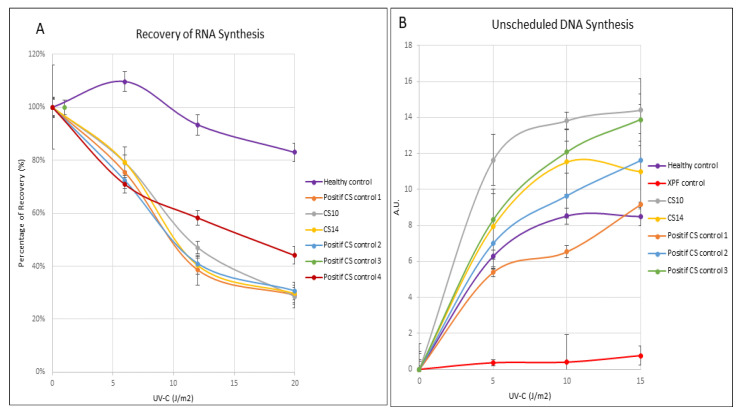
Response to UV radiation in fibroblasts. CS10 and CS14 fibroblasts compared to multiple CS, a XPF, and a healthy control. (**A**) RRS 24h after UV irradiation expressed in percentage of recovery after 5-EU incorporation, showing the defect of RNA Synthesis after UV exposure in CS fibroblasts. (**B**) UDS expressed in arbitrary units (a.u.) of 5-EdU fluorescence intensity. Differently from XP, CS patients show normal levels of unscheduled DNA synthesis.

UV radiation assays tested on fibroblasts derived from CS10 and CS14 patients showed reduced response to UV, compared to healthy controls. The response to increasing doses (0–15 J/m^2^) of UV-C radiation was first assessed by the RRS assay, which showed reduced RNA synthesis in CS10 and CS14 fibroblasts, compared to the healthy control. As expected for CS, unscheduled DNA synthesis UDS levels were normal in CS10 and CS14 fibroblasts. Altogether, these results indicate a defective capacity to repair UV-induced DNA damage on the transcribed strand in two CS patients, including CS14, who does not display clinical hypersensitivity to sunlight (Figure 5).

## 4. Discussion

### 4.1. Common Mutation in Three CS Patients

Cockayne syndrome is a rare autosomal recessive disorder, which is caused by a defect in proteins (CSA or CSB) that are notably involved in the DNA repair system TC-NER. CS, as a form of segmental progeria, recapitulates normal human aging in many of its aspects including cognitive dysfunction, high tone hearing loss, and cataracts [32]. Many autosomal recessive diseases are caused by consanguineous marriages. Due to traditional and cultural customs that incite consanguineous marriages (38% consanguinity), the Tunisian population displays a high frequency of autosomal recessive genetic transmitted pathologies [33]. Indeed, a recent study has identified more than 547 genetic diseases in Tunisia, 60% of which are due to recessive mutations in autosomal genes [34]. 

To date, 17 CS patients have been reported in the Tunisian population [2,16,20,21,22], and the majority of the reported cases (at least 12/17) display genetic defects in the *ERCC8* gene. The relatively larger proportion of *ERCC8* defects in Tunisian patients can be attributed to a probable founder mutation c.598_600delinsAA carrier effect [23].

With this cohort we describe the first *ERCC6/CSB* mutants in the Tunisian population. We reveal a novel frameshift variation c.3156dup, located in exon 18, within three unrelated families belonging to a close geographic area (in northwestern Tunisia). This occurrence suggests a common ancestor, and, therefore, a founder effect, since the three patients were born from first-degree consanguineous parents and exhibited close geographical distribution.

Further investigations, including haplotype analysis, are required to verify a founder mutation in this area. 

### 4.2. The Mutated CSB Protein 

The CSB protein has two major domains: a central ATPase domain, which is composed of an helicase ATP-binding domain spanning from amino acids 519 to 695, and an helicase C-terminal domain, which spans from amino acids 843 to 1002, flanked by N-terminal and C-terminal regions [35].

In addition to its involvement in the TC-NER pathway, the CSB protein, in synergy with CSA, is involved in other cellular processes. Several studies showed the implication of CSB and CSA in the control of p53 levels during genotoxic stress [36], ribosomal biogenesis, [37] proteostasis, cytokinesis [38], mitochondrial DNA repair, [39] and ATF3 degradation [40,41]. Moreover, recent data have shown that CSB plays a master role in replicative senescence of human fibroblasts by unmasking the p21^waf1^ promoter [42]. CSB is also thought to promote p21 degradation through ubiquitylation, due to its E3 ligase activity [43].

A further critical role of CSB C-terminal region in other aspects of the DNA repair process has been highlighted, namely, interaction with RNA polymerase II, translocation of the CSA protein to the nuclear matrix, and association of CSB with chromatin after UV irradiation [44,45,46,47]. 

In the present study, the frameshift variation p.(Arg1053Thrfs*8) was located within the C-terminal region of the CSB protein that interacts with RNA pol II, possibly altering CSB-related transcription. The insertion of an adenosine changed the reading frame and, thereby, the amino acid sequence of the CSB protein. A premature stop codon should then appear eight amino acids from the insertion site, causing the loss of three important regions englobing the C-terminal region that likely contribute to the biological activity of this protein (Figure 3B). The loss of the TC-NER activity was confirmed by reduced RRS and unaffected UDS, upon UV irradiation in two patients, as in standard experiments that demonstrate CSB impairment.

### 4.3. Remarkable Clinical Features and Presence or Absence of Clinical Photosensitivity

The clinical features of CSB patients in the cohort described here tend to be more severe than those reported in North Africa and Middle East, who displayed predominantly mutations in *ERCC8/CSA*. 

Patients in this study were born at term with weight within the low normal range, but showed microcephaly at birth and postnatally. Of note, postnatal height and weight in these patients were lower than those reported in patients with *CSA* mutations at comparable ages and in the same geographical area. For instance, the CS10 patient at 2 years weighted 6 kg and had a height of a 70 cm, which are much lower values than the CS6EA2 patient (*CSA* mutation), namely a weight of 11 kg weight, and a height of 83 cm [23].

Although harboring the same mutation, patients in this study display distinct clinical features of particular interest: one out of three patients in this cohort was not clinically photosensitive. Previous studies reported distinct CS patients that do not present clinical photosensitivity, as in Tunisian, Turkish, Italian, and Moroccan populations, despite the cells of these patients being defective in TC-NER repair [2,16,48,49]. Due to these numerous cases, cutaneous photosensitivity has been classified and is maintained as a minor criterion in the diagnosis of CS. Indeed, several studies have investigated such photosensitivity. It appears in 47% to 76% of cases, with no significant difference between CS patients mutated in *ERCC6* or *ERCC8* [2,3]. As in the previous studies, patients with and without clinical photosensitivity (CS10 and CS14, respectively) displayed impaired RRS and unaffected UDS. These tests confirm that the UV-repair mechanism is indeed impaired in both patients. For this reason, RRS following UV damage remains a useful analysis to confirm the diagnosis and is complementary to genetic investigations.

Pairs of a photosensitive and a non-photosensitive CS patient sharing the same homozygous mutations have been described for the less severe form (type I) for *ERCC6/CSB* (CS22PV/CS28PV) [2] and in our previous study for *ERCC8/CSA* (CS11/CS16) [23] (Supplementary Table S1). Here, we describe one patient associated with the severe CS type II form without photosensitivity (CS14) and two cases (CS10 and CS12) associated with the CS type I form with photosensitivity. Altogether, these data support the notion that clinical photosensitivity requires additional components than the *CSA* or *CSB* mutation. This is probably also the case for other highly heterogeneous CS clinical defects.

### 4.4. Severe Characteristics of the Tunisian CSB Cohort

This cohort displays a certain level of heterogeneity, beyond clinical photosensitivity. For instance, ataxia, cataract, thin skin, pigmentary retinopathy, contractures, neurogenic signs, and teeth abnormalities were alternatively present only in one out the three patients. More specifically, patient CS14 was characterized by severe neuromotor dysfunction, with psychomotor delay as first symptom (at 9 months), and failure to acquire independent sitting and walking capacities. This patient, however did not show ataxia. Patient CS14, who was not clinically photosensitive, displayed pigmentation abnormalities and neurological signs, plus was the only one in this cohort to suffer from optic atrophy. 

Patients CS10 and CS12 displayed growth delay as a first symptom, but the former, differently from the latter, did not display contractures, neurological signs, pigmental retinopathy, dental abnormalities, or thin skin. Conversely, patient CS12 displayed neither hypomyelination (similarly to patients CS10 and CS14), nor cataracts (similarly to patient CS10).

All three CSB patients reported here suffered from lenticular calcifications, brain atrophy, and brainstem, while also showing decreased levels of creatinine in mild serum. A progressive decrease in serum creatinine was reported in the elderly and was suggestive of chronic kidney disease [50]. In addition, all these patients displayed sensory-motor polyneuropathy with a demyelinating mechanism, which involves severe symptoms. Moreover, hearing loss was detected in all reported patients (not shown). In fact, CS is typically associated with prominent sensorineural hearing loss, which is also a frequent age-related condition [37].

To date, neither the type of variation nor its position in the protein have been clearly associated with a specific clinical severity of CS, indicating that underlying mechanisms remain to be elucidated. This situation highlights the requirement of a complete clinical examination, accompanied by genetic analyses, to elucidate complex syndromes such as CS. Several studies have suggested the implication of other mechanisms that may explain specific features of the severe phenotypes, such as neurodegeneration observed in CS patients; for instance, defective transcription of ATF3 responsive genes upon genotoxic stress [41], or alteration of mitochondrial function due to mismanaged oxidative stress [50].

## 5. Conclusions

We identified here a novel homozygous *CSB* frameshift variation that enlarges the mutational and pathological spectra of CS. Patients with this mutation not only display very severe symptoms but also a remarkable level of clinical heterogeneity. In particular, they display (or not) clinical photosensitivity, indicating that this frequent CS defect requires additional factors than the *CSB* mutation to occur. We propose to add this variation to the molecular standard diagnosis of patient clinical features suggestive of CS phenotype, especially for patients originating from northwestern Tunisia and from the North African region. 

## Figures and Tables

**Table 1:** Clinical, biological, imaging, and genetic findings of patients with the novel *CSB* variant.

Family	CS10	CS12	CD14
Sex	M	F	M
Reference sequence	NM_000124.3	NM_000124.3	NM_000124.3
Homozygous ERCC6 mutation	c.3156dup	c.3156dup	c.3156dup
Protein modification	p.(Arg1053Thr*8)	p.(Arg1053Thr*8)	p.(Arg1053Thr*8)
Geographic origin	Northwest	Northwest	Northwest
Consanguinity/Endogamy	Consanguineous	Consanguineous	Consanguineous
Range age at diagnosis (years)	2–5	6–8	6–8
Age at first symptoms(months)	Birth	Birth	9 months
First symptoms	Growth delay	Growth delay	Psychomotor delay
Prenatal abnormalities			
IUGR	NA	NA	NA
Microcephaly	+	+	+
Cerebellar hypoplasia	NA	NA	NA
Oligoamnios	NA	NA	NA
Birth findings			
Birth weight (g)	2550	2300	2950
Birth height (cm)	45	45	48
Head circumference at birth (cm)	33	32	32
Postnatally findings (years)	2–5	6–8	−8
Weight (kg)	6 (−3 SD)	8 (−4 SD)	8 (−3 SD)
Height (cm)	70 (−2 SD)	82 (−6 SD)	76 (−3 SD)
Head circumference (cm)	41 (−2 SD)	40 (−8.5 SD)	42 (−3 SD)
Dysmorphism			
Enophtalmia	+	+	+
Thin skin	−	+	+
Bird-like nose	+	+	+
Neurological findings			
Microcephaly	+	+	+
Psychomotor delay	+	+	+
Independent sitting (months)	8	5	Not acquired
Independent walking (years)	1.5	4	Not acquired
Mental retardation	+	+	+
Limb spasticity	+	+	+
Retractions	−	+	+
Pyramidal signs	+	+	+
Neurogenic signs	−	+	+
Ataxia	+	+	−
Extrapyramidal signs	−	−	−
Epilepsy	−	−	−
Behavioral abnormalities	−	−	−
Ophthalmological findings	+	−	−
Cataract	−	−	+
Optic atrophy	−	+	−
Pigmentary retinopathy			
Otolaryngological findings			
Sensorineural deafness	+	+	+
Auditory evoked response	60/70 dB	60 dB (right ear)/no response for the left ear	NA
Dermatological findings			
Photosensitivity	+	+	−
Eczema	−	−	−
Thin skin	−	+	−
Pigmentation abnormalities	−	−	+
Hair abnormalities	−	−	−
Nail abnormalities	−	−	−
Dental abnormalities			
Caries	+	+	+
Tooth enamel abnormalities	−	+	+
Morphological teeth abnormalities	−	+	+
Laboratory findings			
AST (NV < 40 U/L)	65	108	45
ALT (NV < 40 U/L)	77	180	80
Creatinine (NV50-110 µmol/L)	22	20	31
Imaging findings			
Calcifications	+	+	+
Hypomyelination	+	−	+
Cerebellar atrophy	+	+	+
Brainstem atrophy	+	+	+
Nerve conduction velocities	NA	(SPE. D) 23.5 m/s slowed	(SPE. D) 16 m/s slowed
Neurophysiological findings (ENMG Test)	NA	Sensory and motor demyelinating polyneuropathy	Sensory and motor demyelinating polyneuropathy
Others Findings	Cryptorchidia, toxoplasmosis during pregnancy	−	Cryptorchidia, kyphosis

## Data Availability

All processed data have been provided in the manuscript. Raw data, generated for this study could be provided by the corresponding author upon reasonable request.

## Supplementary Materials

The following are available online at https://www.mdpi.com/article/10.3390/genes12121922/s1, Supplementary Table S1. Presence or absence of clinical photosensitivity in CS patients with the same mutation. Supplementary Figure S1. Growth charts for CS-B patients (0–8 years), compared to the WHO reference charts (mean, 3rd, and 97th percentile). (A) Weight, (B) Height, and (C) Occipital frontal circumference (OFC).
