# Supplementary material for: Identification and Characterization of a Novel Recurrent ERCC6 Variant in Patients with a Severe Form of Cockayne Syndrome B
Source: Genes (Basel). 2021 Nov 29;12(12):1922. doi: 10.3390/genes12121922 (PMC8701866; doi:10.3390/genes12121922)
Supplement: Supplementary file 1 [file genes-12-01922-s001.zip › Supplementary Table S1_Zayoud K.pdf]

Patient code	CS type	Sex	Age at onset	Range age death/last report	Country (origin)	Consanguinity	Gene	Mutation	Protein modification	UV sensistivity	Reported in	Original ref
CS799VI	III			54-57 y (d)	France	suspected	*CSB*	c.544-1G>A/c.2203C>T	p.Glu182Asnfs*4/p.Arg735*	no	Laugel et al, 2010	*Laugel et al, Hum mutat 2010 Feb;31(2):113-26*
CS393VI	III		3 y	19-22 y	France	no	*CSB*	c.544-1G>A/c.1135G>T	p.Glu182Asnfs*4/p.Glu379*	yes	Laugel et al, 2010	*Laugel et al, Hum mutat 2010 Feb;31(2):113-26*
CS683VI	II		0 y	0.5-2 y (d)	USA	no	*CSB*	c.2058G>C/c.2170-1G>A	p.Trp686Cys/p.Val724_Gln762del	no	Laugel et al, 2010	*Laugel et al, Hum mutat 2010 Feb;31(2):113-26*
CS1GL			0 y	0.5-3 y (d)	UK	no	*CSB*	c.2170-1G>A/c.2830-2A>G	p.Val724_Gln762del/p.Ala944Thrfs*1	yes	Laugel et al, 2010	*Laugel et al, Hum mutat 2010 Feb;31(2):113-26*
CS278ST	II	M	0 y	1-3 y	France		*CSB*	c.2060C>T/c.3862C>T	p.Ser687Leu/p.Arg1288*	no	Calmels et al, 2018	*Calmels et al, Orphanet J Rare Dis* 2016;11:26.
CS1GGO	II	M			Germany		*CSB*	c.2060C>T/c.2203C>T	p.Ser687Leu/p.Arg735*	yes	Calmels et al, 2018	*Calmels et al, Orphanet J Rare Dis* 2016;11:26.
CS22PV	I	M	6 m	2-5 y	Italy		*CSB*	c.2096_2097insC/c.2203C>T	p.Leu700Valfs*60/p.Arg735*	no	Calmels et al, 2018	*Calmels et al, Orphanet J Rare Dis* 2016;11:26.
CS28PV	I	M	3 y	2-5 y	Italy		*CSB*	c.2096_2097insC/c.2203C>T	p.Leu700Valfs*60/p.Arg735*	yes	Calmels et al, 2018	*Calmels et al, Orphanet J Rare Dis* 2016;11:26.
CS286ST	I/II	M	0 y	1-4 y	France (Algeria)		*CSB*	c.2599-26A>G/c.4115delG	p.Met867Thrfs*14/p.Gly1372Glufs*2	no	Calmels et al, 2018	*Calmels et al, Orphanet J Rare Dis* 2016;11:26.
CS31PV	II	M	0 y	1-2 y	Pakistan		*CSB*	c.2599-26A>G	p.Met867Thrfs*14	no	Calmels et al, 2018	*Calmels et al, Orphanet J Rare Dis* 2016;11:26.
CS225ST (CS16L0)	I/III	F	2 y	15-17 y	UK		*CSB*	c.466C>T/c.2599-26A>G	p.[Gln156*,Ser142Asnfs*4]/p.Met867	yes	Calmels et al, 2018	*Calmels et al, Orphanet J Rare Dis* 2016;11:26.
4-1			3 y 8 m		Brazil	yes	CSA	c.313_314delGT/c.611C>A	p.Val105Thrfs*6/p.Thr204Lys	no	Laugel et al, 2010	*Laugel et al, Hum mutat 2010 Feb;31(2):113-26*
4-2			9 y 1m		Brazil	yes	CSA	c.313_314delGT/c.611C>A	p.Val105Thrfs*6/p.Val105Thrfs*6	yes	Laugel et al, 2010	*Laugel et al, Hum mutat 2010 Feb;31(2):113-26*
CS15PV	I	M	1.5 y	12- 14 y	Morocco		CSA	c.37G>T	p.Glu13*	no	Calmels et al, 2018	*Laugel et al, Hum mutat 2010 Feb;31(2):113-26*
CS218ST	I	M	0.7 y	11-13 y (d)	India		CSA	c.37G>T	p.Glu13*	yes	Calmels et al, 2018	*Laugel et al, Hum mutat 2010 Feb;31(2):113-26*
CS261ST	I/II	M	0 y	9-11 y	Tunisia		CSA	c.400-2A>G	p.Thr134Leufs*13	no	Calmels et al, 2018	*Calmels N,J Med Genet. 2018 May;55(5):329-343.*
CS133NY	I/III	M	2 y	30-32 y (d)	USA		CSA	c.479C>T (3rd nucleotide before the end of exon 5)	p.Thr134Leufs*13	yes	Calmels et al, 2018	*Calmels N,J Med Genet. 2018 May;55(5):329-343.*
CS11	II	M	0 y	0.5-3 y	Tunisia	yes	CSA	c.598_600delinsAA	p.Tyr200Lysfs*12	no		*Chikhaoui et al, 2021 under review*
CS16	I	F	5 y	5-7 y	Tunisia	yes	CSA	c.598_600delinsAA	p.Tyr200Lysfs*12	yes		*Chikhaoui et al, 2021 under review*

Supplementary Table S1. Presence or absence of clinical photosensitivity in CS patients with the same mutation. CS patients with identical mutations in one or both alleles**.** M: male; F: female. Mutations in two alleles are separated by "/". Cases of identical mutations in both alleles are undescored in grey. (d) years at death. Empty boxes indicate unavailable data.
