# Supplementary figures and images for: Identification and Characterization of a Novel Recurrent ERCC6 Variant in Patients with a Severe Form of Cockayne Syndrome B
Source: Genes (Basel). 2021 Nov 29;12(12):1922. doi: 10.3390/genes12121922 (PMC8701866; doi:10.3390/genes12121922)

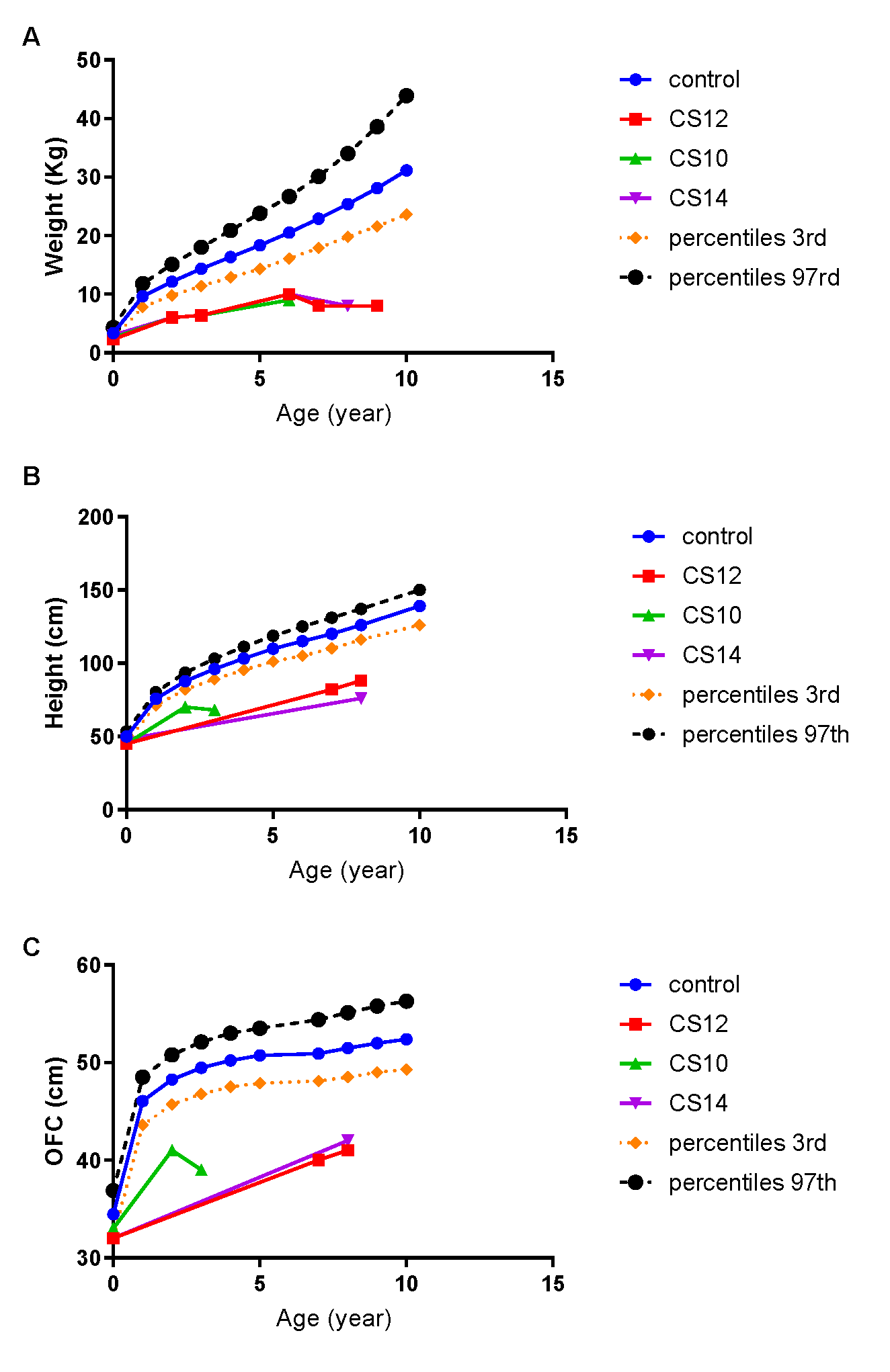

Supplement: Supplementary file 1 [file genes-12-01922-s001.zip › Supplementary Figure S1_Zayoud K.tif]
